# Bacterial Acyl Carrier Proteins Are a Cytoplasmic Target for Different Cationic Antimicrobial and Antibiofilm Peptides

**DOI:** 10.3390/ijms27114823

**Published:** 2026-05-27

**Authors:** Gopal Ramamourthy, Subrata Paul, Ishrat M. Jalal, Hiroaki Ishida, Hans J. Vogel

**Affiliations:** Biochemistry Research Group, Department of Biological Sciences, University of Calgary, Calgary, AB T2N 1N4, Canada; rgopal@ucalgary.ca (G.R.);

**Keywords:** acyl carrier protein, *Francisella novicida*, *Pseudomonas aeruginosa*, antimicrobial peptides, antibiofilm peptides

## Abstract

Cationic antimicrobial peptides (AMPs) that can target multidrug-resistant pathogenic bacteria via multiple mechanisms are considered promising alternatives to antibiotics. Small (~9 kDa) highly acidic acyl carrier proteins (ACPs), which are a well-known cofactor protein in bacterial fatty acid synthesis (FAS), are a potential intracellular target for AMPs. A previous study has demonstrated that the human AMP LL-37 can bind to ACP and thereby affect FAS and the bacterial membrane integrity. In this work, we have investigated the interactions of different classes of AMPs and antibiofilm peptides (ABPs) with the ACPs of two pathogens. We first studied the folding characteristics of the two ACPs and found that *Pseudomonas aeruginosa* ACP (PaACP) is fully folded at neutral pH in the absence of divalent cations. On the other hand, the homologous *Francisella novicida* ACP (FnACP) is unfolded at low ionic strength, but it adopts a fully folded conformation after the addition of divalent cations such as Ca^2+^ or Mg^2+^. These distinct characteristics were shown to be related to a unique His residue that is involved in a stabilizing cation–π interaction. Subsequent biophysical SPR and NMR interaction studies reveal that cationic AMPs and ABPs such as LL-37, melittin, tritrpticin, indolicidin, puroindoline A, lactoferricin B and IDR-1018, but not F5W-magainin 2, can bind to both apo- and holo-ACPs. Binding of Arg-rich peptides is preferred over their Lys-rich analogs. Interestingly, all the peptides bind to holo-ACP with higher affinity than to apo-ACP, which lacks the functionally important phosphopantothenate group. NMR peak intensity perturbation data reveal that helix II of ACP, which is known to be directly involved in complex formation with bacterial FAS enzymes, acts as a common and main recognition site for the peptides. We propose that binding of AMPs and ABPs to this region of bacterial ACPs can directly block fatty acid synthesis and interfere in other ACP-dependent biosynthetic and regulatory events, which in turn could contribute to killing the bacteria and could also intervene in biofilm formation.

## 1. Introduction

Due to the rapid emergence of antibiotic-resistant bacterial pathogens and the limited number of new antibiotics that are currently in the drug pipeline, there is an urgent need for the development of novel antibiotics to combat these infections [1,2]. Antibiotic resistance has become a global health threat, and the efficacy of conventional antibiotics has been further compromised by biofilm-forming bacteria. Disease-causing microorganisms that are encased in a biofilm are highly resistant to multiple drugs [3], and they are responsible for many chronic and long-term infections [4].

Since their initial discovery in the 1980s, antimicrobial peptides (AMPs) have been evaluated as promising alternatives for today’s antibiotics [5]. AMPs are ubiquitous in nature and they are found in most microorganisms, plants, and animals [6]. Many AMPs are classified as host-defense peptides because they also have a role in immunomodulation as part of the natural host defenses [7]. The presence of several cationic amino acids and a significant proportion of hydrophobic residues are common features of most AMPs. These attributes promote membrane interactions of AMPs with the negatively charged phospholipid headgroups of the microbial membranes of bacteria and provide selectivity over the zwitterionic headgroups of the phospholipids that make up the outer leaflet of mammalian membranes [8,9].

AMPs have a remarkable capacity to kill pathogens and many of them have a broad spectrum of activities, including antibacterial, antifungal, antiviral, and anticancer effects [10]. When acting on bacteria, the mode of action of AMPs can be broadly categorized into two groups. One is the lytic mechanism where the AMPs participate in cell lysis by disrupting the cell membrane [11]. A second mechanism is non-lytic, where AMPs can rapidly permeate the cell membrane and then interfere with essential intracellular processes. Evidence suggests that several AMPs have intracellular targets such as DNA, RNA, and heat shock chaperone proteins (DnaK, GroEL); however, the precise mechanism of cell membrane permeation for these AMPs remains elusive [12].

Two models for the non-lytic mechanisms have been proposed: one is the spontaneous translocation of AMPs, and the other is the bacterial protein-assisted translocation [1,13,14]. According to the spontaneous translocation model, AMPs can participate in transient pore formation in the bacterial membrane due to their amphipathic nature. Such AMPs can briefly disrupt the lipid chain association of the cytoplasmic cell membrane, and when the pore falls apart, the AMPs can translocate to the inside of the cell [15]. As such, most lytic peptides also end up inside the bacterial cell after some time, where they can do additional damage beyond the original membrane perturbations. Conversely, a number of proline-rich AMPs are known to be internalized directly into Gram-negative bacteria through specific bacterial membrane transporter proteins [14].

It has been demonstrated that different AMPs can affect membrane septum formation and cell wall synthesis, inactivate essential intracellular enzymes, and inhibit DNA, RNA or protein synthesis [12]. Recently, Hoek et al. reported that the acyl carrier protein (ACP) from *Francisella novicida* could be a potential target for the human cathelicidin AMP LL-37. They also proposed a broader action of LL-37-ACP binding as LL-37 was also shown to be involved in complex formation with the ACPs of *Escherichia coli* and *Bacillus anthracis* [16]. These intriguing data stimulated us to further investigate the binding of a wide variety of structurally diverse AMPs to ACPs from two different pathogenic bacteria, *F. novicida* (FnACP) and *Pseudomonas aeruginosa* (PaACP). We have selected the ACPs of these two well-known pathogens for further study, in part, because their folding characteristics had not previously been characterized.

*F. novicida* serves as a surrogate organism to study infections caused by the potential bioterrorism agent, *Francisella tularensis* [17]. Biological safety level-3 containment is required for research with *F. tularensis* due to its low infectious dose and high mortality rate. Although *F. novicida* shares 97% overall genomic identity with *F. tularensis*, *F. novicida* appears to be non-pathogenic to humans and hence it is often used as a model organism [18,19]. *P. aeruginosa* is a well-known pathogen that is responsible for infections of different organisms including animals and humans. This opportunistic pathogen is one of the most common causes of nosocomial infections including pneumonia, bloodstream infections, urinary tract infections, and surgical site infections in patients with compromised host defenses [20]. Moreover, both of these Gram-negative pathogens can form biofilms and thereby become virtually resistant to multiple conventional antibiotics [21,22,23,24].

The small highly acidic ~9 kDa ACP (also known as AcpP) plays a central role in bacterial fatty acid synthesis (FAS) by shuttling the growing acyl chain between the catalytic centers of the various enzymes that make up the FAS system [25]. Closely related carrier proteins also have a pivotal role in bacterial secondary metabolism such as the biosynthesis of gramicidin S, erythromycin, vancomycin, and the siderophore enterobactin through non-ribosomal peptide synthetase and polyketide synthase systems [26]. Before the acylation reaction on ACP can occur, the enzyme acyl carrier protein synthase (AcpS) utilizes Coenzyme-A to posttranslationally modify a specific Ser sidechain at a DSL sequence of ACP with a phosphopantothenate group. This changes the inactive apo-ACP into the active holo-ACP. The 20 Å long phosphopantetheine (PPant) arm tethers the growing fatty acyl intermediates through a reactive thioester linkage [27]. In bacterial ACPs, the fatty acid hydrocarbon chain once attached to the PPant arm, becomes inserted in the core of the protein [28]. ACP is central to the FAS biosynthetic system and its interaction with partner enzymes is critical for the fatty acid biosynthesis [25]. ACP is often a highly abundant protein in bacteria, for example, it contributes 0.25% of all proteins in *E. coli* [26].

Fatty acid synthesis is one of the most conserved cellular processes and is essential for bacterial membrane biosynthesis, making FAS an attractive target for drug discovery [29]. FAS not only contributes to membrane integrity and fluidity [30] but ACP is also associated with the biosynthesis of lipoic acid [31] and biotin [32]. FAS-ACP also participates in quorum sensing by synthesizing signaling molecules such as homoserine lactones [33] and quinolones [34]. Furthermore, FAS has been implicated for a role in the fatty acid modification in virulence factors such as staphyloxanthin [35] and hemolysin [36] biosynthesis. Differences in the organizations of eukaryotic and bacterial FAS may enable researchers to design selective inhibitors for the bacterial pathway [37]. Large multifunctional multidomain proteins constitute the mammalian type I FAS [38], whereas bacterial type II FAS consists of a set of discrete monofunctional enzymes [39]. Intensive studies are ongoing to develop new tools to interfere in the ACP-partner protein interactions inherent to the bacterial pathway. Most of these inhibitors are designed to target FAS enzymes [40,41,42,43], and two of these compounds (AFN-1252, Triclosan) have already shown encouraging results in human clinical trials [44,45].

To date, efforts to develop a novel AMP that can be used to circumvent the antibiotic resistance, including tackling the multidrug-resistant pathogens, have been partially thwarted by the lack of a detailed molecular understanding of the role of AMPs in intracellular processes. Although it has been reported that the acidic bacterial ACP can be a cytoplasmic target for the human α-helical AMP LL-37 [16], the molecular details of this binding event remain elusive. Therefore, in this work, we have undertaken a more comprehensive and structural study. First, we used a label-free approach, Surface Plasmon Resonance (SPR)-mediated screening, to investigate the binding and determine the dissociation constants for a wide variety of AMPs with different chemical features and distinct structural properties. Most cationic AMPs are unstructured in aqueous solution, but they usually become structured when binding to a target. While nearly all cationic AMPs can form amphipathic structures when bound to a membrane or a membrane-mimetic surface, they do so with different secondary structures [1]. Thus, we studied representatives from the α-helical class (LL-37, melittin, F5W-magainin 2), the Trp-rich turn structure class (tritrpticin, indolicidin, puroindoline A) as well as the β-hairpin class (lactoferricin B). The amino acid sequences of the peptides used in this work are listed in Table 1, and additional information about the properties of these peptides is provided in Table S1. Several of these peptides are also known to have antibiofilm activities [46,47]. Moreover, we have also studied the antibiofilm peptide (ABP) IDR-1018, which has very limited lytic antimicrobial activity but strongly inhibits biofilm formation nonetheless [48]. After completely assigning the backbone amide groups along the protein sequence in the ^1^H,^15^N HSQC NMR spectra of the *F. novicida* and *P. aeruginosa* ACPs, we could conduct NMR chemical shift and peak intensity perturbation titration experiments to identify the AMP and ABP binding sites on the ACPs of these two Gram-negative organisms.

## 2. Results

### 2.1. Multiple Sequence Alignment

**Figure 1:**
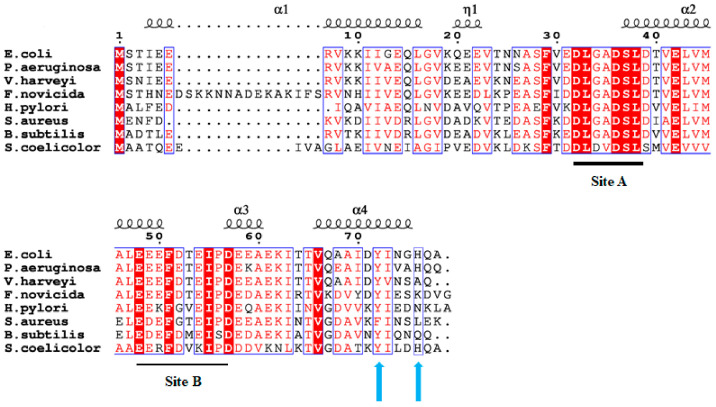
Amino acid sequences of acyl carrier proteins involved in fatty acid synthesis from *Escherichia coli*, *Pseudomonas aeruginosa*, *Vibrio harveyi*, *Francisella novicida*, *Helicobacter pylori*, *Staphylococcus aureus*, *Bacillus subtilis*, and *Streptomyces coelicolor* are aligned. The known divalent cation binding sites in *E. coli* ACP (A and B) are indicated. Residues aligned with position His75 and Tyr71 of *E. coli* ACP are highlighted by blue arrows. A secondary structure diagram of FAS-ACP from *E. coli* is provided above the amino acid sequences. Clustal Omega (1.2.4 version) was used for multiple sequencing alignment. In addition, for the visualization and annotation of sequence alignments, particularly with secondary structure information, the ESPript 3 software version 3.2 tool was used.

We have carried out an amino acid sequence alignment of bacterial ACPs, which are known to be involved in fatty acid synthesis in various Gram-negative and Gram-positive microorganisms. The sequence alignments shown in Figure 1 revealed that all the ACPs had a very high conservation of anionic residues. In a previous study concerning the ACPs from *E. coli* and *Vibrio harveyi* [49], we have shown that these two clusters of anionic residues constitute two binding sites for the divalent metal ions, Ca^2+^ and Mg^2+^. The sequence alignment indicates that all ACPs have these two binding sites positioned in the same location. It should be noted that the sequences of the ACPs from the three Gram-positive organisms (*S.a*, *B.s* and *S.c*) align well with those of the other Gram-negative organisms. We also observed an unexpected insertion of twelve residues near the N-terminal end of FnACP that is not present in the proteins from the other bacteria. Interestingly, the sequence alignment revealed the presence of a Lys residue at position 91 in FnACP near the C-terminal end, whereas a His is found at the homologous position 75 for the *E. coli* and *P. aeruginosa* proteins (Figure 1).

### 2.2. Folding Studies of ACPs

**Figure 2:**
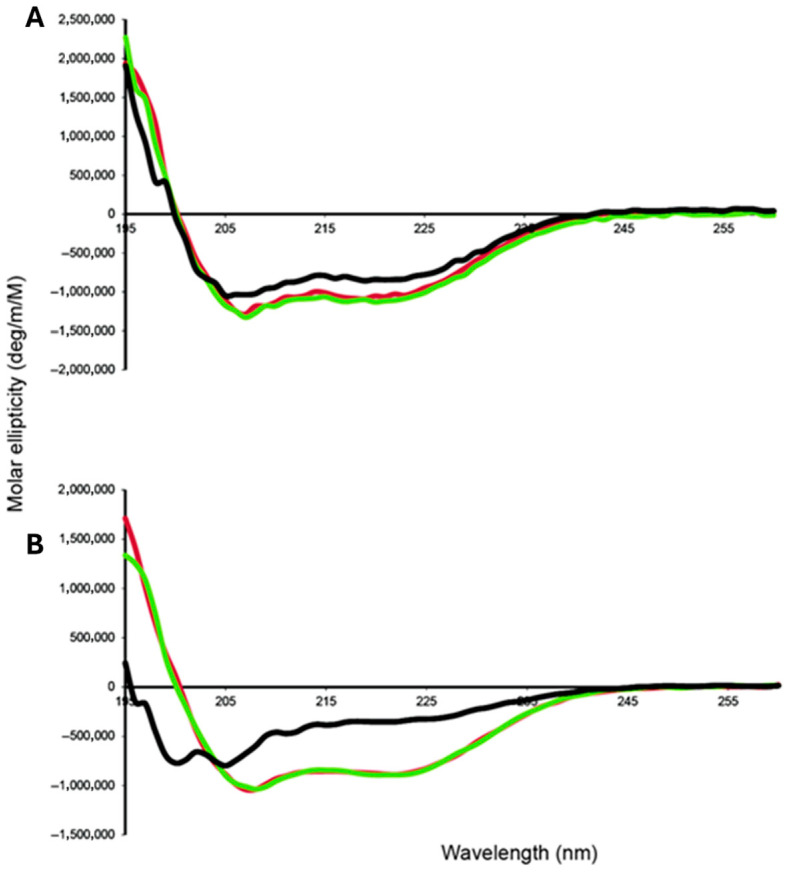
CD spectra of PaACP (**A**) and FnACP (**B**) in the presence of 5 mM HEPES buffer at pH 7.0. Each panel shows the spectral overlay of ACP with EDTA (Black), MgCl_2_ (Green), and CaCl_2_ (Red).

CD spectroscopy has been used to initially characterize the global folding of the ACPs. In particular, we have characterized the effects of the addition of divalent cations on the structure and conformation of the ACPs. The CD spectra of PaACP recorded at low ionic strength in the presence of EDTA, MgCl_2_ or CaCl_2_ at pH 7.0, showed that under all conditions the protein has an α-helical structure as revealed by the characteristic double dip negative features at 208 nm and 220 nm in the CD spectra, as well as the positive feature at 195 nm (Figure 2A). When compared to the CD spectra for PaACP recorded in the presence of divalent cations, the PaACP-EDTA CD spectrum displayed a further decrease in the negative intensity of the 208 nm and 220 nm bands, suggesting that the addition of the divalent cations to the PaACP causes further stabilization of the alpha helices (Figure 2A). In contrast, the CD spectrum of FnACP at low ionic strength did not show a proper helical structure in the absence of divalent cations and it suggests the presence of an unfolded protein (Figure 2B). Upon addition of MgCl_2_ or CaCl_2_, the CD spectrum of FnACP indicated the presence of a folded conformation with a large alpha-helical content (Figure 2B). DSC measurements show that the Ca^2+^ and Mg^2+^ forms of FnACP denature at 75 °C and 73 °C, respectively, which is similar to the value measured for Ca^2+^-EcACP at 73.3 °C (while apo-EcACP denatures around 52.7 °C [49]).

**Figure 3:**
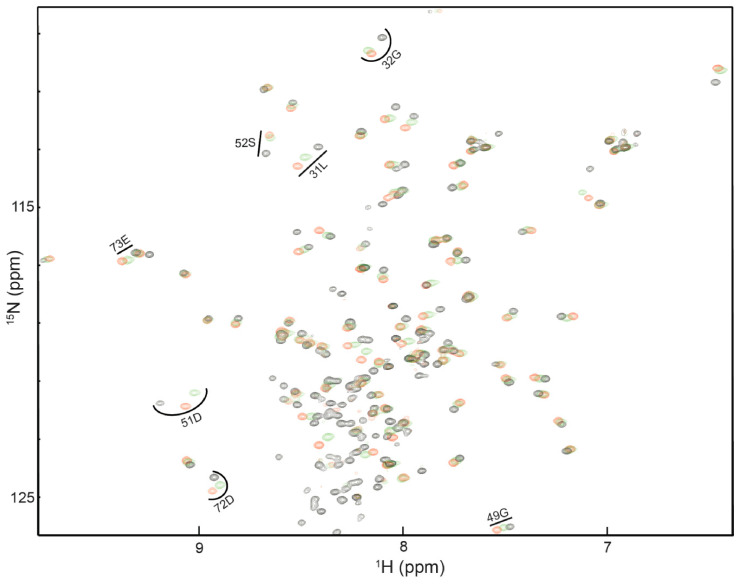
^1^H,^15^N HSQC NMR spectra of ACP from *F. novicida* were overlaid for folding comparison in the presence of 2 mM EDTA (Black), 10 times excess of MgCl_2_ (Green), and CaCl_2_ (Red).

**Figure 4:**
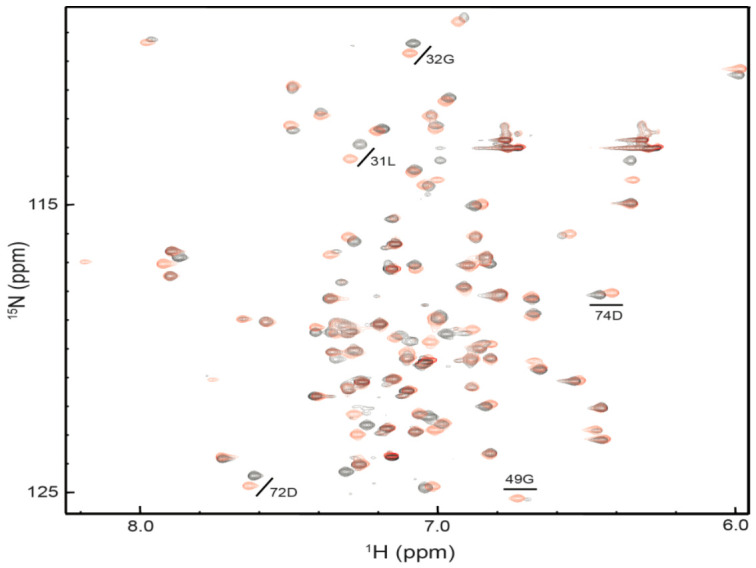
Overlay of ^1^H,^15^N HSQC NMR spectra from wild type FnACP in the presence of 10 times excess CaCl_2_ (Red) and the K91H mutant of FnACP in the absence of CaCl_2_ (Black).

We also carried out initial ^1^H, ^15^N HSQC NMR experiments to determine the folding status of FnACP in relation to the presence of the divalent cations (Figure 3). Our data shows that FnACP in a buffer containing 100 mM NaCl, was partially unfolded in the presence of EDTA compared to the Ca^2+^ and Mg^2+^ bound forms (Figure 3). We also observed in titration studies that the addition of Ca^2+^ ions had a larger effect on the chemical shifts than Mg^2+^. Spectra for both the Ca^2+^- and Mg^2+^-bound FnACP showed the typical features of a folded protein (Figure 3). Based on the HSQC NMR and the amino acid sequence alignment data, we investigated whether the substitution of Lys91 with His could cause changes in the FnACP conformation in EDTA. Similar to what was observed for the *V. harveyi* ACP A75H mutant [49], the ^1^H,^15^N HSQC NMR spectrum of the K91H variant of FnACP, acquired at low ionic strength, revealed that this single amino acid substitution induced a fully folded conformation in the protein (Figure 4).

**Figure 5:**
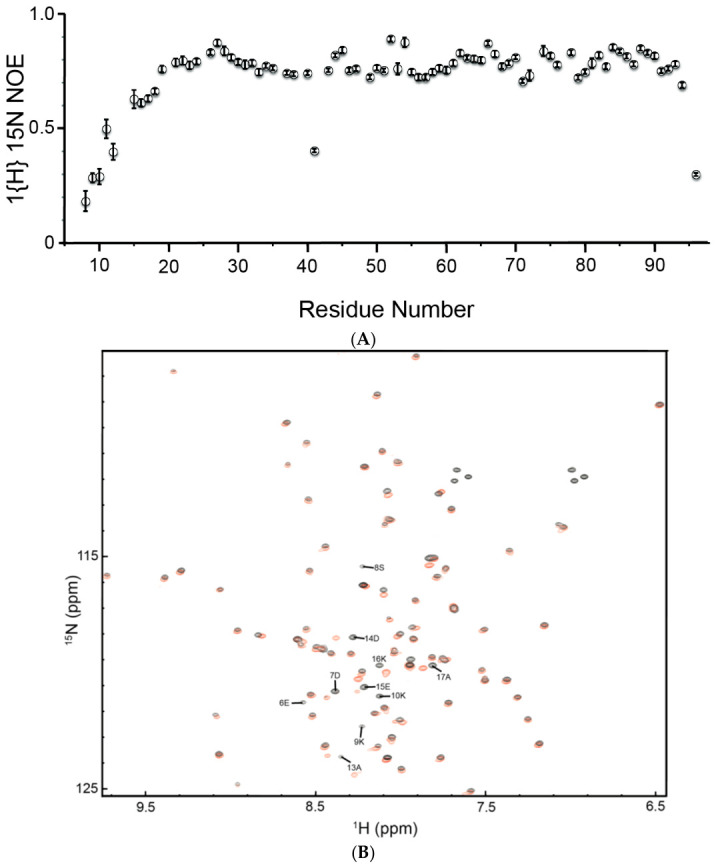
(**A**). The backbone {^1^H},^15^N-NOE data for apo-FnACP, where each residue is represented by open circles and the vertical error bars represent the standard deviation. The low NOE value (~0.45) for one of the resonances in the loop between helix I and helix II may be due to an unexpected exchange phenomenon. (**B**). An overlay of the ^1^H,^15^N HSQC NMR spectrum of wild type FnACP (black) with that of truncated FnACP (red) with a deletion of the first 12 residues. Residues at the N-terminal end of the wild type FnACP are highlighted.

In order to gain some insights into the 12-residue N-terminal extension of FnACP, we performed an NMR dynamics experiment. These heteronuclear ^15^N NOE relaxation data indicate that the first ~15 residues of this protein are flexible, compared to the remainder of the protein, which behaves as expected for a small globular protein (Figure 5A). Furthermore, we have checked the structural preservation of the truncated form of FnACP, in which the first 12 amino acids of the protein were deleted. Clearly, the ^1^H,^15^N HSQC spectrum of truncated FnACP almost completely overlaid with the NMR resonances originating from the wild type FnACP except for those of the twelve residues of the N-terminal deletion. The latter peaks were found to be clustered around the middle of the HSQC spectrum of the full-length protein (Figure 5). These data indicate that truncated FnACP retains a correctly folded conformation without the aforementioned N-terminal residues of the wild type FnACP. Moreover, the chemical shift data also indicate that a large part of the 12-residue insertion is not folded into a regular secondary structure.

### 2.3. SPR Based Binding Assay

Since the structural characterization of FnACP and PaACP revealed that the presence of divalent cations is important for their stability and proper folding, we carried out all our binding assays in a buffer containing a saturating amount of CaCl_2_. The S52A mutant of ^15^N-apo FnACP and the ^15^N-holo FnACP (wild type) (Supplemental Figure S1) were used for this study. We preferred the use of the S52A mutant of ^15^N-apo FnACP over ^15^N-apo FnACP (wild type), as the purification of the homogenous S52A mutant protein of ^15^N-apo FnACP proved to be easier than that of ^15^N-apo FnACP (wild type). In the case of PaACP, we mostly used the wild type versions of ^15^N-apo PaACP and ^15^N-holo PaACP, as both these forms of PaACPs could be readily separated, and their nature and purity could be confirmed by NMR spectroscopy (Supplemental Figure S2).

**Figure 6:**
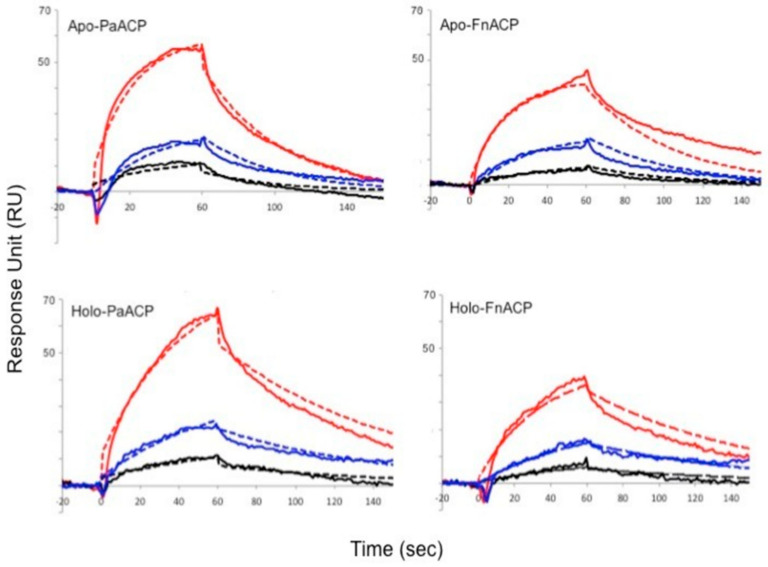
Representative SPR data obtained for the binding of IDR-1018 to apo- and holo-PaACP (**left** panel) and FnACP (**right** panel). Sensorgrams and fittings are shown in solid and dashed lines, respectively. Three different concentrations of IDR-1018 are represented by three different colors.

We carried out SPR screening assays to determine whether selected AMPs display high-affinity binding to PaACP and FnACP. To this end, we immobilized the N-terminal cysteine-tagged ACP versions from FnACP and PaACP on two different CM chips. Antimicrobial peptides including LL-37, melittin, F5W-magainin 2, tritrpticin, indolicidin, puroindoline A and lactoferricin B were then allowed to interact with the ACPs, and the data obtained are summarized in Table 2. The results showed that nearly all AMPs tested interacted with both forms of ACPs (apo and holo) and bound with dissociation constants in the low micromolar range. Additionally, we also observed that these peptides had a higher affinity for the holo-ACPs than for their apo-counterparts (Table 2 and Figure 6). The ABP IDR-1018 was also tested in the SPR measurements and was shown to bind to all proteins as well (Table 2). To ascertain that the values obtained by SPR under our experimental conditions were reasonable, we also performed an ITC (isothermal titration calorimetry) experiment with apo-PaACP and IDR-1018, which provided a Kd value of 2.8 µM, in close agreement with the SPR results.

Our SPR data suggested that the cytotoxic α-helical melittin peptide, the major component of bee venom, is bound to the apo- and holo- forms of FnACP and PaACP with the highest affinity compared to the other AMPs under our experimental conditions. On the other hand, no binding was observed for the Lys-rich F5W-magainin 2 peptide. While LL-37 appeared to display strong binding to apo-FnACP (1.33 μM), we observed a slightly weaker affinity of LL-37 for apo-PaACP (8.43 μM). In the case of the holo-ACPs, LL-37 interacted with an affinity that is close to the data obtained for the interaction of melittin with holo-ACPs (Table 2).

The SPR studies also demonstrated that both the AMP tritrpticin and the ABP IDR-1018 bound to both apo-ACPs with very similar affinities. Moreover, both these peptides appeared as tight binders towards the holo-ACPs compared to their apo counterparts. Two other Trp-rich AMPs, indolicidin and puroindoline A, as well as the 25-residue disulfide crosslinked β-hairpin peptide lactoferricin B seemed to be relatively weaker binders for both apo- and holo-FnAPCs. In parallel, apo-PaACPs were also shown to associate with indolicidin, puroindoline A and lactoferricin B. Interestingly, the dissociation constants obtained for holo-PaACPs were comparable to the data obtained for melittin binding to holo-PaACP (Table 2) and they support the general trend that the affinity of the peptides for holo-ACPs is higher than for apo-ACPs.

### 2.4. NMR-Derived Binding Data

**Figure 7:**
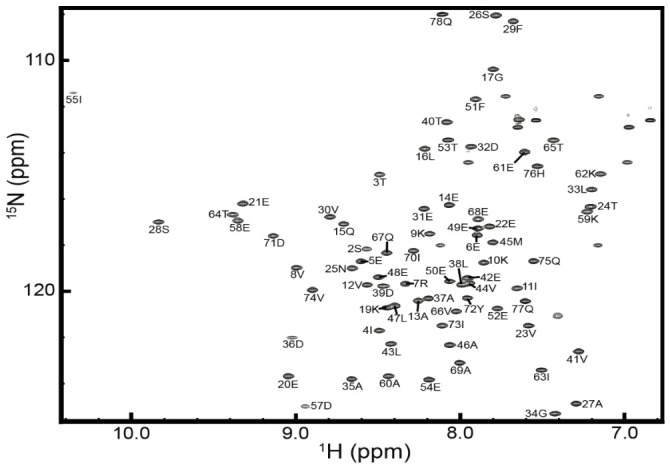
The fully assigned ^1^H,^15^N HSQC NMR spectrum of folded apo-ACP from *P. aeruginosa*. The spectrum was acquired at 25 °C in the presence of 20 mM HEPES, 100 mM NaCl, and 10 times excess CaCl_2_.

**Figure 8:**
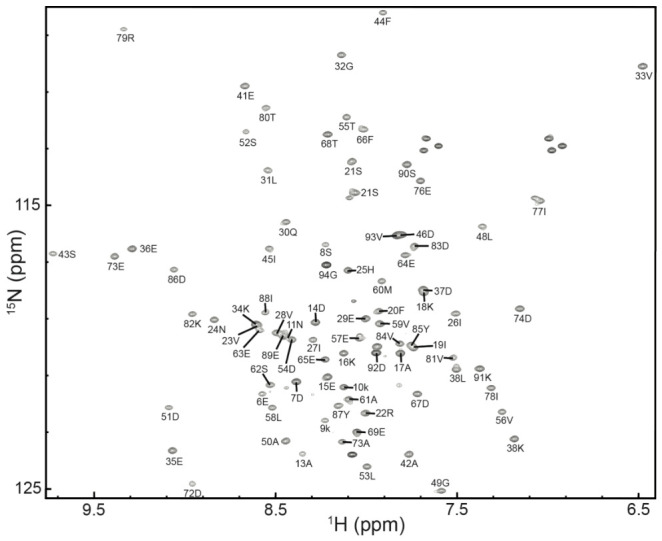
The fully assigned ^1^H,^15^N HSQC NMR spectrum of folded apo-ACP from *F. novicida*. The spectrum was acquired at 25 °C in the presence of 20 mM HEPES, 100 mM NaCl, and 10 times excess CaCl_2_.

The ability to track changes in the chemical shifts of the peaks or to follow changes in the intensities of the peaks in the NMR spectra makes it possible to map out binding sites on ACPs when AMPs are added during a titration experiment. To facilitate this works, the chemical shifts of the backbone resonances for both apo-PaACP (S37A) and holo-FnACP needed to be determined. This was achieved using routine 3D NMR assignment strategies, and the backbone amide assignments for these two proteins are indicated in Figure 7 and Figure 8, respectively. As can be seen, the backbone assignments are complete for more than 95%, as can typically be achieved for such relatively small proteins by these NMR methods [49,50].

**Figure 9:**
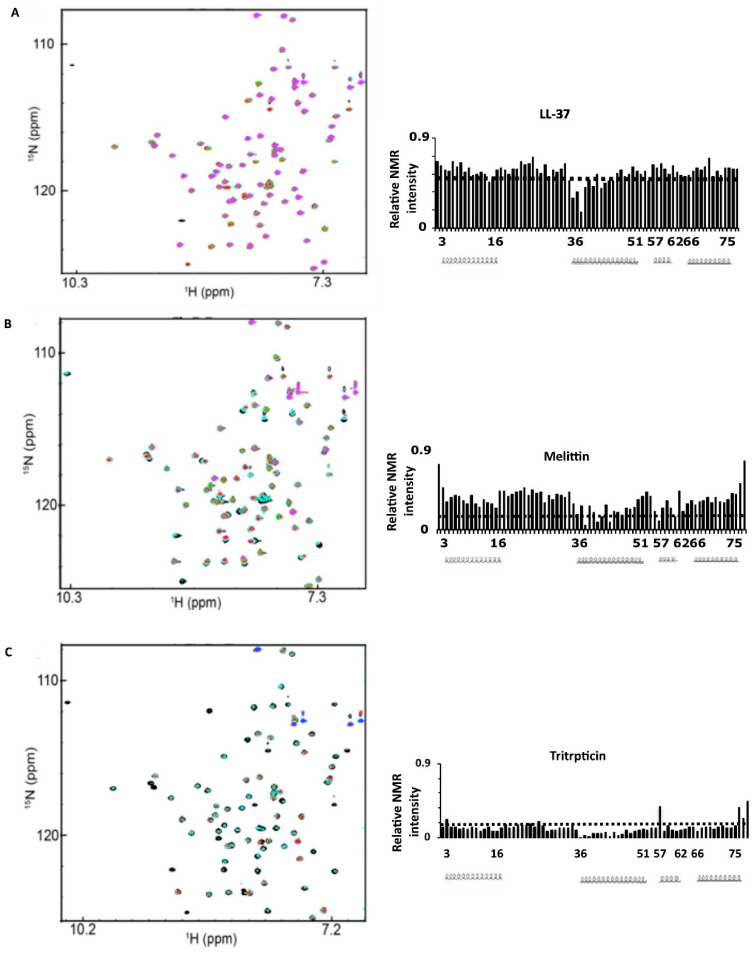

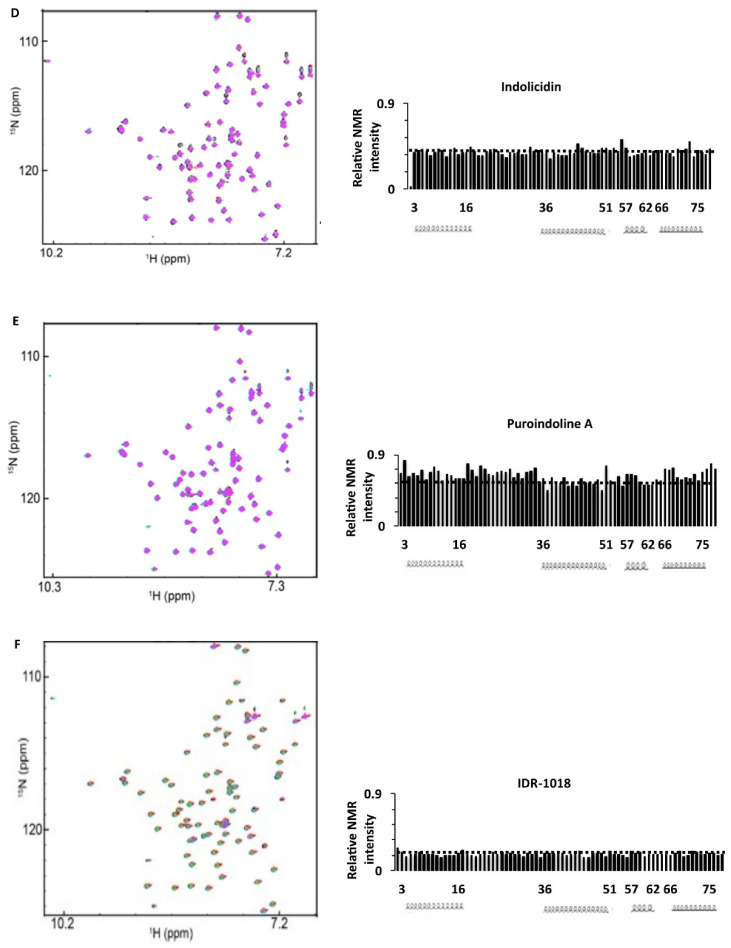
NMR titration data recorded for the apo-ACP from *P. aeruginosa* in the presence of excess LL-37 (**A**), melittin (**B**), tritrpticin (**C**), indolicidin (**D**), puroindoline A (**E**), and IDR-1018 (**F**). Throughout each representative data apo-PaACP appears in black, whereas 0.2, 0.4, 0.6, 0.8, and 1.0 times excess AMPs are shown in red, green, blue, cyan, and magenta, respectively.

**Figure 10:**
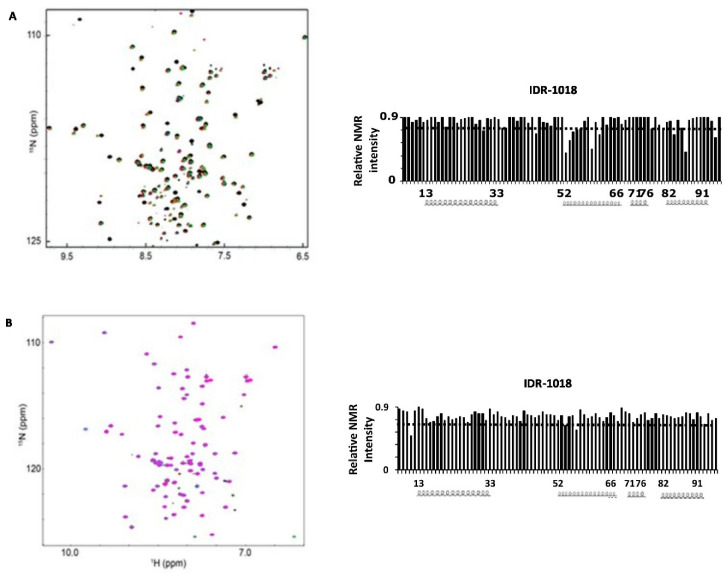
NMR titration data recorded for the holo-ACP from *F. novicida* in the presence of excess IDR-1018 (**A**) and for the S52A apo-ACP from *F. novicida* in the presence of excess IDR-1018 (**B**). For each representative data ACP appears in black, whereas 0.2, 0.4, 0.6, 0.8, and 1.0 times excess AMPs are shown in red, green, blue, cyan, and magenta, respectively.

With the backbone assignments completed we initiated titration experiments of all the peptides with both proteins. The results of these experiments are shown in Figure 9 and Figure 10.

In general, no or very limited chemical shift changes were observed, but rather changes in the peak intensities were observed, in nearly all cases. The latter changes can be explained by two processes. First, binding of the peptides to specific regions of the protein in “intermediate exchange” would lead to selective broadening of the NMR resonances for the protein residues that are directly involved in the interaction with the peptide. Secondly, the binding of peptides seemed to cause aggregation of the proteins, which would give rise to broadening of all the resonances at the same time, leading to a general reduction in all NMR peak intensities. In most of the titrations, both of these events seem to occur simultaneously, as both general and selective peak intensity reductions are seen, but to differing degrees depending on the peptide (and or the protein). The fact that no chemical shift changes and only specific peak broadening is observed is consistent with the low micromolar affinities obtained in the SPR data, which typically give rise to intermediate exchange NMR conditions. Weaker binding would lead to so-called “fast exchange” conditions, which would be seen as chemical shift changes, which were not detected. Likewise, there is no evidence for stronger binding, as this would be observed as the appearance of new NMR peaks, reflecting the so-called “slow exchange” NMR conditions. Unfortunately, intermediate exchange behavior, generally makes it more difficult to perform more detailed NMR analyses, such as structural determinations. However, from the selective broadening effects seen, we can still conclude where the peptides bind to the surface of the proteins. Going over the NMR titration data for all the peptides, it is clear that residues along helix II of the protein are frequently affected, in particular those near the N-terminal part of this helix, where the PPant group becomes attached. However, other residues on helix I and Helix III are sometimes also affected, and this differs between the peptides.

Two other notions can also be deduced from these data. First of all, we did not observe any chemical shift changes or intensity losses in the ^1^H,^15^N HSQC NMR spectra of apo-PaACP and apo-FnACP during titrations with the Lys-rich F5W-magainin 2 peptide (Figure S3). This result is consistent with the SPR data and confirms that this peptide does not bind to the protein. Secondly, in titrations of the other peptides with the FnACP protein, no changes were observed for the resonances representing the first 12 residues of the protein. Hence, it seems unlikely that this flexible region of the protein plays a major role in the binding of the AMPs and ABPs. Finally, while the intensity changes for the PaACP and FnACP are not identical, in general the same highly negatively charged surface of the proteins appears to be involved in the binding of the peptides.

**Figure 11:**
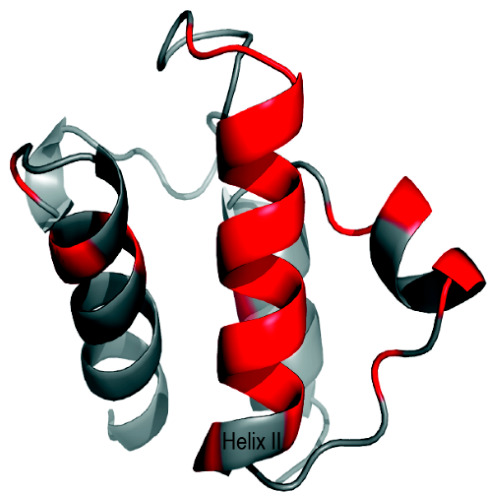
Residues (red) of ACPs involved in the interaction with melittin and various AMPs (Supplementary Figure S4) are mapped on the model structure of apo-ACP from *P. aeruginosa* FAS system. The SWISS-MODEL homology program was used to build the model using the solution structure of *V. harveyi* (2L0Q) as a template.

In order to more clearly identify the specific residues in apo-PaACP that make up the binding interface for these AMPs (Figure 11 and Supplementary Figure S4), we have modeled the structure of PaACP using the solution structure (2L0Q) of FAS ACP from *V. harveyi* as a template (which has 81% sequence identity). Subsequently, we have mapped the residues which experienced lower intensities during the titration with the peptides on the structure. Residues such as V12, E14, K19, E20, A35, D36, A37, L38, D39, T40, V41, E42, L43, V44, M45, A46, L47, E50, I55, D57, E61, K62, I63, and I70 were all associated with intensity losses to varying degrees. Out of the 24 amino acids that were affected, 13 amino acids reside in helix II of apo-PaACP. The other affected residues are on the same face of the protein and involve parts of helices I and III. It can also be seen that most of the residues around the DSL site for the attachment of the PPant moiety are all affected by peptide binding (residues 35–47).

## 3. Discussion

### 3.1. Structural Characterization

The CD spectroscopy data have shown that *F. novicida* ACP adopts an unfolded conformation at low ionic strength and in the absence of divalent ions. On the other hand, PaACP is fully folded in the presence of either EDTA or divalent cations (Figure 2). In this respect, PaACP behaves similarly to *E. coli* ACP, which is fully folded under these conditions. However, the *F. novicida* ACP resembles the *V. harveyi* ACP (VhACP) as both these proteins require divalent cations to adopt a folded conformation in low ionic strength buffers [49]. To identify the binding sites of the divalent ions in both PaACP and FnACP, we have compared the amino acid sequences of seven bacterial ACPs from *Escherichia coli*, *Pseudomonas aeruginosa*, *Vibrio harveyi*, *Francisella novicida*, *Helicobacter pylori*, *Bacillus subtilis*, *Staphylococcus aureus* and *Streptomyces coelicolor*. These data clearly indicate that all these ACPs contain two conserved divalent cation binding sites that are positioned on either side of the helix II region (Figure 1). Occupation of these sites by Ca^2+^ or Mg^2+^ leads to structure stabilization, which in turn can give rise to a drastic increase in the thermal denaturation temperature by ~20 °C [49].

We also noted that FnACP has another similarity to VhACP, namely that both proteins do not have a conserved His near their extreme C-terminal end (Figure 1). It was previously demonstrated that the A75H mutant of VhACP at low ionic strength adopts a folded conformation in the absence of divalent ions, whereas the wild-type VhACP is unfolded under these conditions, as is the *H. pylori* ACP [49]. Likewise, when the regular His-75 residue of *E. coli* ACP was mutated to Ala, the EcACP became unfolded whereas the wild type EcACP protein is properly folded [51]. As was observed for the A75H mutant of VhACP, the K91H mutant of FnACP could fully restore the folded conformation (Figure 4) at low ionic strength in the absence of any divalent cations. The side chain of the His-75 residue in EcACP and VhACP-A75H has an unusually high pKa and is positively charged at pH 7.0; as such, it can participate in a cation–π stacking interaction with the totally conserved aromatic Tyr-71 residue. This interaction contributes to the stabilization of helix IV in these proteins [49]. The critical cation–π interaction cannot form when the His is not present, leading to an ACP that is unstable under low ionic strength conditions [51,52]. Our current data further highlight the importance of this particular cation–π interaction for the stabilization of the structures of bacterial FAS-ACPs.

In view of the fact that a conserved His at this position seems like an essential feature, we can now try to predict whether a bacterial FAS-ACP will be folded or unfolded in the absence of divalent cations. In Figure S5, we have compared the amino acid sequences of the ACPs from the ESKAPEE pathogens that are responsible for most hospital-acquired bacterial infections [53]. Based on these alignments, we can hypothesize that the ACPs from bacterial pathogens such as *S. aureus*, *A. baumanni* and *E. faecium* will also be unfolded in the absence of Ca^2+^ or Mg^2+^ at lower ionic strength, as they also lack the important His residue. Future research will have to determine whether this structural instability in some ACPs has any impact on the virulence of these organisms. Be that as it may, the concentration of Mg^2+^ inside most bacterial cells is believed to be sufficient to saturate, at least partially, the two metal-binding sites of the ACPs in vivo [28,52].

Our CD spectroscopy data show that the proteins have a mostly alpha-helical structure. The NMR backbone assignment data can provide additional information about the secondary structure of the protein. In the case of calcium-saturated PaACP and FnACP, we could observe in the NMR data the formation of the four alpha-helices that are typically seen for the folded bacterial FAS-ACPs. Further 3D structure determination was not pursued here, as structures can now be reliably predicted for the ACP helix bundle with Alphafold-3 modeling [54,55], and our main interest was in studying the AMP binding. However, before doing so, we have also characterized the properties of the unique 12-residue insertion (Figure 1) at the N-terminus of FnACP. Our ^15^N-heteronuclear NOE NMR data (Figure 5A) revealed that these additional residues are rather flexible. Moreover, their chemical shifts are consistent with an unstructured region. Consequently, we can conclude that these residues do not interact directly with the typical four-helix bundle conformation of the bacterial ACPs. To further test this hypothesis, a construct was expressed with the 12 N-terminal residues deleted. The ^1^H,^15^N HSQC NMR spectral overlay of this truncated FnACP with the wild type FnACP (Figure 5) revealed that the vast majority of their NMR resonances overlap, indicating that the helix bundle of the protein is still correctly folded and that these 12 residues are not essential for the structural integrity of the protein. Nonetheless, future biochemical studies are required to further examine the biological role, if any, of these unique additional residues in FnACP.

### 3.2. Interaction Studies of ACPs with AMPs and ABPs Using SPR and NMR

In recent in vitro studies, LC-MS/MS experiments, bead pull-down assays and differential scanning fluorimetry were used to show that the AMP LL-37 can bind to the ACPs from *F. novicida*, *E. coli* and *B. anthracis* [16]. Thus, the highly negatively charged cytoplasmic bacterial ACP was identified as one of the intracellular targets for the human cationic AMP LL-37. Furthermore, the authors showed that the fatty acid composition of the bacterial *F. novicida* cells was perturbed following administration of LL-37, suggesting that binding and inhibiting bacterial ACPs by AMPs contributes to the membrane perturbation and bactericidal activity of LL-37 in this strain [16]. These authors also suggested that ACPs from other pathogenic microorganisms such as *P. aeruginosa* and *Burkholderia pseudomallei* might be an intracellular target of LL-37 as well. In the present study, we have used different experimental techniques (SPR and NMR), to demonstrate that LL-37 interacts with the Ca^2+^-bound ACPs from *P. aeruginosa* and *F. novicida*. The positively charged AMP LL-37 can bind to the polyelectrolyte ACP through electrostatic interactions (pI of FnACP = 4.23; pI of PaACP = 4.07). Even after binding two equivalents of Ca^2+^ or Mg^2+^, the bacterial ACPs still carry a very high negative overall charge. Indeed, it is well known that LL-37 can bind to extracellular targets that are negatively charged molecules such as LPS and polysaccharides, and thereby promote anti-inflammatory activity and antifungal activity against *C. albicans* infections [56,57]. Another study has suggested that the LL-37:DNA complex contributes to the host defense antimicrobial activity against intracellular bacteria in human macrophages [58]. Clearly, highly cationic AMPs, such as LL-37 and related cationic peptides can utilize relatively long-range electrostatic interactions to attract and then bind to highly negative charged polyelectrolytes, including the bacterial membranes, LPS and nucleic acids (DNA and RNA), for example.

In the case of the membranolytic peptide melittin [59] our SPR and NMR studies also provided evidence for melittin-binding to ACP. This result is consistent with previously published data that also indicated that *E. coli* ACP can interact with melittin and that this can lead to inhibition of fatty acid synthesis [60]. Another study also demonstrated that the acidic mammalian protein calmodulin (pI 4.3) has high affinities for many basic antimicrobial peptides, including LL-37 and melittin, but not for F5W-magainin 2 [61]. In contrast to the two helical AMPs LL-37 and melittin, we did not observe any chemical shift perturbations or intensity losses during the NMR titrations between apo-PaACP (S37A) and F5W-magainin 2 (Figure S3), which is a helical Lys-rich peptide (Table 1). However, when we performed such NMR titrations with an analog of this peptide in which the three Lys of F5W-magainin 2 were substituted with Arg, some minor intensity losses and small chemical shift changes were observed for apo-FnACP and apo-PaACP indicating that binding occurs, albeit weakly. It is possible that the guanidinium group of Arg can enhance the interactions of AMPs with the negatively charged surfaces of ACPs because of its more directional hydrogen bonding potential compared to Lys. It is worth noting that high Arg contents have been noted in many of the target proteins for ACPs [62]. Furthermore, the guanidinium group of Arg can form favorable cation–π interactions with aromatic amino acids, which could further explain the preference of ACPs for binding Arg-rich peptides.

For the three Trp-rich AMPs tritrpticin, indolicidin and puroindoline A, we found that the SPR data indicate that tritrpticin binds with higher affinity than the other two, which could be related to its relatively higher Arg content. We also observed binding for these peptides to both PaACP and FnACP. These three peptides are much shorter than LL-37 and melittin and they all have a turn-like structure when bound to membranes or membrane mimetics (Table S1). While our current NMR data do not allow us to deduce the secondary structure of the ACP bound AMP, we note that several AMPs with a high propensity to form α-helices or turn-structures can all bind to the bacterial ACPs investigated here. This suggests that the peptide–protein interaction does not require a specific secondary structure for the bound peptide. Several recent studies have reported that Trp-rich AMPs do not only act on bacterial membranes but can also perturb various intracellular bacterial targets, such as the negatively charged biomolecules DNA and RNA and various proteins, which contributes to their bactericidal activity [63,64,65,66,67]. Our data indicate that ACPs can be added as one of the proteins on this “target” list, which would provide an additional mechanism of action for the AMPs for killing of bacterial cells.

With respect to the β-hairpin lactoferricin B peptide, we note that it binds with similar avidity as the other peptides according to the SPR data. However, during NMR titrations, which require much higher protein and peptide concentrations than the SPR studies, extensive precipitation was observed, and while this indicates binding, it does not allow us to draw conclusions about the binding site of this structurally constrained peptide. While most of the other peptides also led to aggregation in protein–peptide complexes, these complexes remained mostly soluble.

Finally, the ABP IDR-1018, which is not lytic and does not perturb anionic bacterial membranes [68] also binds to the ACPs. It is thought that this peptide can translocate across the membrane and then perturb intracellular targets to achieve its antibiofilm activity. We note that IDR-1018 is of similar length as the three Trp-rich peptides, and that it is also an Arg-rich peptide (Table 1). Intriguingly, it also has a very high affinity for ACPs (Table 2), which is consistent with the notion that Arg-rich peptides are preferred over Lys-rich peptides for binding to ACPs. A recent study suggests that IDR-1018 interferes in bacterial metabolism during the so-called “stringent response” and that it can target the small highly negatively charged secondary messenger (p)ppGpp [69] that is essential for coordinating bacterial physiological responses during biofilm formation [70]. On the other hand, other researchers indicated that (p)ppGpp itself is perhaps not a specific target for IDR 1018 [71]. Clearly, based on our data, ACP presents itself as an alternative intracellular target for this ABP that is involved in (p)ppGpp metabolism. In this regard, it is important to point out that bacterial ACPs are known to bind to and regulate the activity of the SpoT enzyme. The latter is responsible for the “stringent response”, by synthesizing the regulatory nucleotide (p)ppGpp during nutrient stress [72,73,74]. This unique bacterial regulatory nucleotide is involved in increasing biofilm formation and antibiotic resistance, amongst various other metabolic effects. Thus, AMPs and ABPs potentially can in this fashion interfere in (p)ppGpp synthesis and its associated actions. Interestingly, helix II on ACP has been identified as the binding site for SpoT [75], which overlaps with the binding site identified here for the AMPs and ABPs.

Our data suggests that holo-ACPs are stronger binding partners for the AMPs and ABPs than their apo-counterparts (Table 2) irrespective of the bacterial strains of ACPs. The presence of the PPant group on the conserved (DSL) serine contributes an additional negative charge near the protein surface through a phosphodiester linkage [53], which is likely responsible for the higher affinity of the cationic AMPs towards holo-ACPs. This phosphodiester linkage could partially explain the preference for Arg-rich peptides, as the guanidinium group of Arg is known to provide very strong and specific interactions with such phosphate-containing linkages [76,77].

Numerous experiments and modeling studies have indicated the importance of “recognition” helix II of ACP in the bacterial FAS system [78,79,80,81], that mediates fatty acid biosynthesis in bacteria. Our NMR data suggested that helix II of PaACP and FnACP is the primary site of interaction for many cationic AMPs and that this interaction would block this enzyme binding site on the ACPs. Some peptides also interact with the residues in helix I and III that are part of the same surface that surrounds helix II on the protein (Figure 11 and Figure S4). Interestingly, several hydrophobic residues of helix II were also perturbed during titration with the AMPs, suggesting that short-range hydrophobic and van der Waals interactions could also play a role in the binding, in addition to the electrostatic interactions (Figure 11).

As the amino acid sequences of FAS-ACPs are highly conserved, (Figure 1) it follows that residues from ACPs involved in the interaction with FAS enzymes will be very similar, if not identical. Available information for such interactions has been summarized in Table S2. Based on this analysis, we can hypothesize that the majority of the AMPs used in our studies could interfere with the activity of the bacterial FAS system. As fatty acids are an integral component of the cell membrane, perturbation of this enzyme activity could provide an additional mechanism for lethality for bacterial pathogens [16]. Previous in vivo studies with an ACP that was blocked by introducing a nonreactive pantothenamide group resulted in the disruption of fatty acid synthesis and ultimately cell death [82,83]. Moreover, a recent study has identified inhibitors for the bacterial ACP-synthase enzyme as a potential novel class of antibiotics [84], which would block the generation of holo-ACP from apo-ACP, and would give rise to very similar effects as can potentially be achieved by the binding of AMPs and ABPs to helix II of the ACPs.

As mentioned above, most AMPs do not have a single mechanism of action. In a recent review paper, cationic AMPs have been referred to as “dirty drugs” that can hit numerous targets [15], both in the bacterial (inner and outer) membranes as well as several highly negatively charged intracellular targets such as DNA, RNA, ACP and other proteins. These actions could take place simultaneously or sequentially, depending on the chemical properties of the peptide. For example, melittin is known to give rise to strong membrane depolarization and this means that this can dominate its killing action, while any effects caused by its binding to intracellular targets such as ACP would likely be minimal. On the other hand, IDR-1018 acts mostly inside the cell, and hence ACP binding could be very important, but this peptide would cause some membrane disruption as well, as it enters the cell. The other peptides studied here fall in between these two extremes and many of them would rely on a combination of different membrane perturbation and intracellular effects to achieve bacterial killing. Further contributing to this complexity is the fact that many AMPs have been shown to cause changes in gene expression or act as antibiofilm agents at sublethal concentrations [4,15]. Hence, further biological studies are required for each individual peptide to determine how much AMP/ABP binding to ACPs contributes to their overall bactericidal or antibiofilm effect.

## 4. Materials and Methods

### 4.1. Cloning, Expression and Protein Purification

Codon-optimized genes for the ACPs from *P. aeruginosa* and *F. novicida* were purchased from GeneArt (Regensburg, Germany). The genes were subcloned with 5′ *NdeI* and 3′ *XhoI* restriction sites into a pET15b expression vector containing an N-terminal hexa-histidine tag followed by a Tobacco Etch virus (TEV) protease cleavage site. Additionally, the genes were mutated in order to generate apo-ACPs with an added N-terminal Cys residue by using site-directed mutagenesis in the presence of appropriate primers. Site-directed mutagenesis was also carried out to create an alanine variant (Asp-Ala-Leu) of the serine residue from the highly conserved Asp-Ser-Leu motif of both ACPs to avoid the biosynthetic generation of a mixture of apo- and holo-ACPs during expression in *E. coli*. Moreover, a gene truncation strategy was carried out to generate an N-terminal deletion variant of FnACP where the first twelve residues of the protein were deleted. An additional point mutation was carried out to substitute Lys91 of FnACP with a His for further characterization of the folding properties of the *F. novicida* protein. All ACP gene constructs were verified through nucleotide sequencing at the University of Calgary sequencing facility. All the verified plasmids were transformed into competent *E. coli* strain BL21 (DE3) for protein expression.

*E. coli* cells containing the vector of interest were grown in Luria broth (LB) media, supplemented with 100 μg/mL ampicillin at 37 °C. Uniformly isotope-labeled ^15^N- and ^15^N,^13^C-proteins were prepared by protein expression in M9 minimal media containing 0.5 g/L ^15^NH_4_Cl and 3 g/L ^13^C_6_-glucose (or unlabeled glucose). The culture containing the desired fusion protein was grown to mid-log phase (A_600_~0.6) at 37 °C and induced with 0.5 mM isopropyl β-D-1-thiogalactopyranoside (IPTG). After 20 h of additional incubation, the cells were harvested by centrifugation at 6000× *g* for 15 min.

The cell pellets were resuspended in lysis buffer (50 mM Tris/HCl, 100 mM NaCl, 40 mM imidazole, pH 8.0) and lysed by three passes through a French press. The cell lysate was then centrifuged at 18,000× *g* for 45 min. If necessary, the clarified supernatant was converted to holo-ACP by incubating with coenzyme A in the presence of crudely purified *E. coli* AcpS [85]. A pre-equilibrated Ni-affinity column (GE-Healthcare, Mississauga, ON, Canada) was used to purify both apo- and holo-ACPs. The column was washed extensively with lysis buffer (50 mM Tris/HCl, 100 mM NaCl, 40 mM imidazole, pH 8.0) and the fusion protein was eluted with elution buffer (50 mM Tris/HCl, 100 mM NaCl, 300 mM imidazole, pH 8.0). The eluted protein fractions were detected by A_280_ and pooled together for overnight dialysis in 20 mM Tris/HCl, 100 mM NaCl pH 8.0 at 4 °C. After addition of 0.5 mM EDTA and 5 mM 2-mercaptoethanol, the fusion protein was subjected to TEV-protease digestion at room temperature for 3 h. The removal of the His-tag after TEV protease cleavage resulted in the addition of three residues (GHM) to the N-terminus of the ACPs. TEV protease was expressed and purified from the pRK793 plasmid (Addgene) as previously described [86]. The digested sample was applied to the O-complete (GE-Healthcare) column to remove the cleaved histidine tag. The protein was then dialyzed (3 times) with 10 mM Tris/HCl pH 7.0 and further purified by using a Resource Q anion exchange column with stepwise NaCl gradients from 0 to 1 M. The purity of the protein was confirmed by sodium dodecyl sulfate polyacrylamide gel electrophoresis (SDS-PAGE) and the concentration of the protein was determined using the extinction coefficient at 280 nm obtained theoretically from the ExPASy-ProtParam program (2017 version). Some samples were mixed with 2× sample buffer (300 mm Tris, pH 6.8 and 60% glycerol) and were analyzed by 12% PAGE without SDS.

### 4.2. Circular Dichroism Spectroscopy (CD Spectroscopy)

The lyophilized proteins were dissolved in 5 mM HEPES buffer in the presence of either 2 mM EDTA or 2 mM CaCl_2_ or 2 mM MgCl_2_, 1 mM DTT at pH 7.0. The CD spectra were recorded from 260 nm to 195 nm at a slow scanning speed in a 0.1 cm quartz cell at room temperature with a Jasco J-810 spectropolarimeter (Easton, MD, USA). The experiments were performed at a protein concentration of 10 μM at least three times, where each experiment was composed of 10 accumulations.

### 4.3. Surface Plasmon Resonance Spectroscopy (SPR)

A BIAcore X100 instrument (GE Healthcare, Chicago, IL, USA) was used for the SPR experiments to evaluate the binding between ACPs and different AMPs. For these experiments, the ACP variant (Cys-ACP) with an extra N-terminal Cys residue was used. These proteins were expressed and purified according to the protocol stated in the previous section. The majority of the peptides used here were purchased as synthetic peptides with >95% purity from Genscript (San Diego, CA, USA). Melittin was obtained from Sigma Aldrich (Oakville, ON, Canada).

A CM5 sensor chip (GE Healthcare) was used to immobilize Cys-ACP via thiol coupling. The running buffer contained 20 mM HEPES, 150 mM NaCl, 1 mM CaCl_2_, and 0.005% (*v*/*v*) Tween-20. Five concentrations of the peptide solutions were prepared for each cycle starting at 10 μM and continuing with 3-fold dilutions after. Peptides were injected at a flow rate of 30 μL/min with a contact time of 1 min at 25 °C. SPR sensorgrams were processed by using the BIAevaluation software 2.0 (GE Healthcare) and dissociation constants (*K_D_*) were obtained by curve-fitting the data.

### 4.4. Differential Scanning Calorimetry (DSC)

The denaturation temperatures of some of the proteins were determined by DSC, which was performed as described in our previous studies with bacterial ACPs [49].

### 4.5. NMR Spectroscopy

All NMR experiments were performed at 298 K on Bruker Avance 500 MHz or 700 MHz spectrometers (Bruker, Billerica, MA, USA). ^15^N or ^15^N,^13^C-labeled apo-, holo-, or truncated ACPs were prepared in 20 mM HEPES, 100 mM NaCl, pH 7.0. An additional 2 mM dithiothreitol (DTT) was added only to the NMR samples of ACPs with an N-terminal cysteine. All the NMR experiments were carried out in the presence of 10% D_2_O, 0.5 mM 2,2-dimethyl-2-silapentane-5-sulfonic acid (DSS), and 0.03% sodium azide. The sequential backbone resonance assignments of both apo-ACPs were obtained using various three-dimensional NMR experiments including HNCACB, CBCA(CO)NH, HNCO, HN(CA)CO, HNCA, and HN(CO)CA. The NMR chemical shift assignments of ^1^H and ^15^N for apo-Fn ACP were used to map the chemical shifts obtained from the ^1^H,^15^N HSQC NMR spectra of the truncated- and K91H-mutated FnACP variants.

{^1^H}-^15^N Heteronuclear NOE data for the backbone dynamics studies of ^15^N-apo FnACP were acquired at the ^15^N-frequency by using a 5 s train of 120^0^ proton pulses. Two dimensional ^1^H,^15^N HSQC NMR experiments were acquired by adding either LL-37, melittin, F5W-magainin 2, tritipticin, indolicidin, puroindoline A, lactoferricin B and IDR-1018 in ~0.3 mM ^15^N-isotope labeled apo- or holo-PaACPs samples. Two dimensional ^1^H,^15^N HSQC NMR experiments were carried out for apo-PaACP and FnACP in the presence of either CaCl_2_ or MgCl_2_ or EDTA in a buffer containing 20 mM HEPES, 100 mM NaCl at pH 7.0. All spectra were processed and analyzed by the NMRPipe [87] and NMRView [88] software (2015 version).

## 5. Conclusions

**Figure 12:**
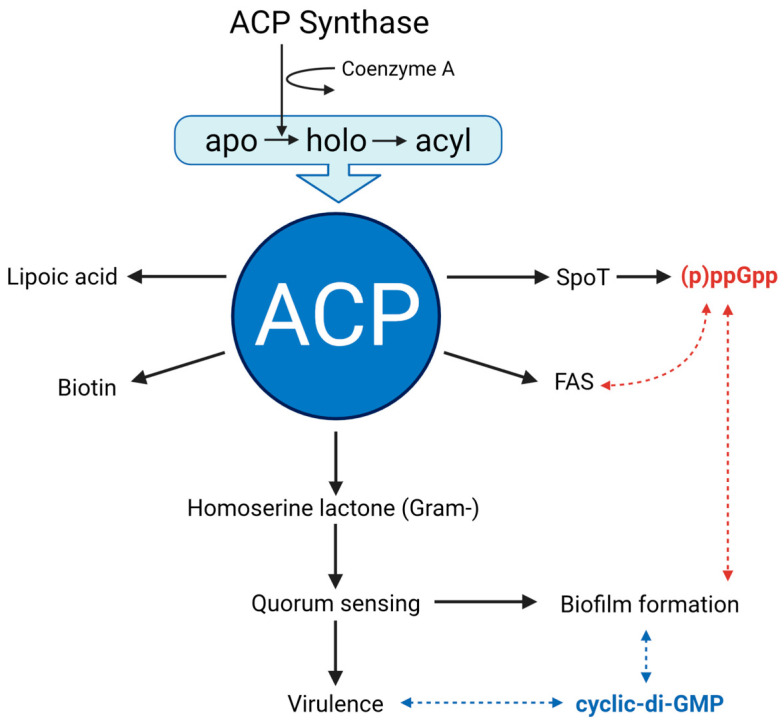
Schematic diagram showing the central role of ACP in various metabolic reactions and the production of regulatory signaling nucleotides in bacteria.

Taken together, our data indicates that many, but not all, cationic AMPs and ABPs are able to interact with the cytoplasmic bacterial FAS-ACPs with micromolar affinities. Arg-rich peptides seem to bind more tightly than those containing multiple Lys residues, while the secondary structure propensities of the peptides seem less important. NMR analysis revealed that the majority of the AMPs bind mostly to the highly negatively charged “recognition” helix II of the bacterial ACP, preferentially near its N-terminal end where the DSL site and the site A for binding divalent cations are located. The binding of AMPs to helix II and the surrounding surface of the ACPs would block the formation of productive enzyme complexes, which would inhibit the fatty acid synthesis in bacteria. In turn, this would alter the bacterial membrane composition [16,60]. Additionally, because ACPs are involved in various other biosynthetic activities, the binding of the cationic AMPs and ABPs could also interfere with bacterial lipoic acid, biotin and quorum sensing biosynthesis, thereby further compromising bacterial virulence. Furthermore, binding of AMPs to the ACPs, could directly affect its binding to the SpoT enzyme, and thereby intervene in the production of the bacterial intracellular secondary messenger (p)ppGpp, which is normally involved in regulating biofilm formation and metabolic shifts during environmental stress. Figure 12 provides a schematic overview highlighting the central role of ACP in these bacterial metabolic and regulatory events. In this diagram we have also indicated the role of cyclic-di-GMP, another regulatory nucleotide that is intimately involved in bacterial exopolysaccharide and biofilm formation [89]. The synthesis of both these regulatory nucleotides is interdependent and hinges on the level of GTP in Gram-negative bacteria such as *P. aeruginosa* [90].

Finally, we note that the location of the main binding site for the AMP and ABPs is in close proximity to the conserved DSL site for PPant attachment at the N-terminal end of the ACP helix II. This would suggest that binding of AMPs to the apo-form of bacterial ACPs could block the attachment of the functionally important phosphopantothenate group by the ACP synthase enzyme. Binding of AMPs/ABPs would then give rise to accumulation of an inactive ACP in cells, which would inhibit bacterial growth [84]. Future work could concentrate on this aspect by measuring the enzymatic activity of ACP synthase in the presence of AMPs. Moreover, it could also be of interest to study the binding of AMPs to ACPs in the absence of the divalent metal ions. Given the even higher overall net negative charge of these proteins in the absence of the divalent cations, one would expect that the binding of the AMPs and ABPs would be stronger because of increased electrostatic interactions between the cationic peptides and the highly acidic protein.

## Figures and Tables

**Table 1:** Amino acid sequences of all AMPs/ABPs used in this study.

AMPs	Sequence
LL-37	LLGDFFRKSKEKIGKEFKRIVQRIKDFLRNLVPRTES
Melittin	GIGAVLKVLTTGLPALISWIKRKRQQ
F5W-magainin 2	GIGKWLHSAKKFGKAFVGEIMNS-NH_2_
Tritrpticin	VRRFPWWWPFLRR-NH_2_
Indolicidin	ILPWKWPWWPWRR-NH_2_
Puroindoline A	FPVTWRWWKWWKG-NH_2_
Lactoferricin B	FKCRRWQWRMKKLGAPSITCVRRAF
IDR-1018	VRLIVAVRIWRR-NH_2_

**Table 2:** Represents the binding data of AMPs with both forms of *F. novicida* and *P. aeruginosa* ACPs. Dissociation constants are obtained from SPR measurements in the presence of 10 mM HEPES, 150 mM NaCl, 1 mM CaCl_2_ and 0.01% Tween-20 at pH 7.0. In this table “-” sign indicates no binding.

AMPs	Apo-FnACP (μM)	Holo-FnACP (μM)	Apo-PaACP (μM)	Holo-PaACP (μM)
LL-37	1.33	0.66	8.43	0.38
Melittin	1.07	0.64	1.38	0.38
F5W-Magainin 2	-	-	-	-
Tritrpticin	1.39	0.64	1.56	1.12
Indolicidin	6.85	3.4	12	0.56
Puroindoline A	183	3.6	1.81	1.03
Lactoferricin B	10.7	5.5	3.23	0.83
IDR-1018	2.25	0.68	2.39	0.94

## Data Availability

Data are contained within the article. Further inquiries can be directed to the corresponding author.

## Supplementary Materials

The following supporting information can be downloaded at: https://www.mdpi.com/article/10.3390/ijms27114823/s1.
