# Supplementary material for: Bacterial Acyl Carrier Proteins Are a Cytoplasmic Target for Different Cationic Antimicrobial and Antibiofilm Peptides
Source: Int J Mol Sci. 2026 May 27;27(11):4823. doi: 10.3390/ijms27114823 (PMC13256389; doi:10.3390/ijms27114823)
Supplement: Supplementary file 1 [file ijms-27-04823-s001.zip › ijms-4282485-supplementary.pdf]

Supplementary file

Bacterial acyl carrier proteins are a cytoplasmic target for different cationic antimicrobial and antibiofilm peptides

Gopal Ramamourthy[+], Subrata Paul[+], Ishrat M. Jalal, Hiroaki Ishida, and Hans J. Vogel*

Biochemistry Research Group, Department of Biological Sciences, University of Calgary, Calgary, AB

T2N 1N4, Canada; rgopal@ucalgary.ca (G.R.);

* Correspondence: vogel@ucalgary.ca; Tel.: +1-403-220-6006; Fax: +1-403-289-9311.

[+]Gopal Ramamourthy and [+]Subrata Paul contributed equally to this work

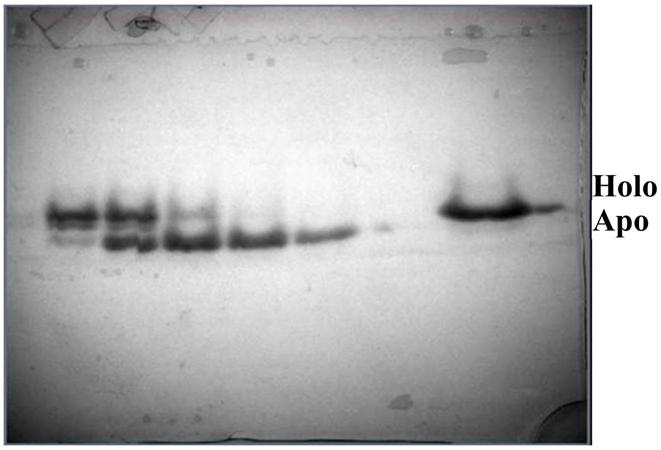

Figure S1. Native gel of FnACP (wild type) fractions obtained during purification of the protein showing that holo-FnACP can be purified with good yields in the later fractions. On the other hand, the earlier fractions, containing the majority of apo-FnACP, also include the holo-protein and some undigested protein.

Apo-PaACP
(wild type)

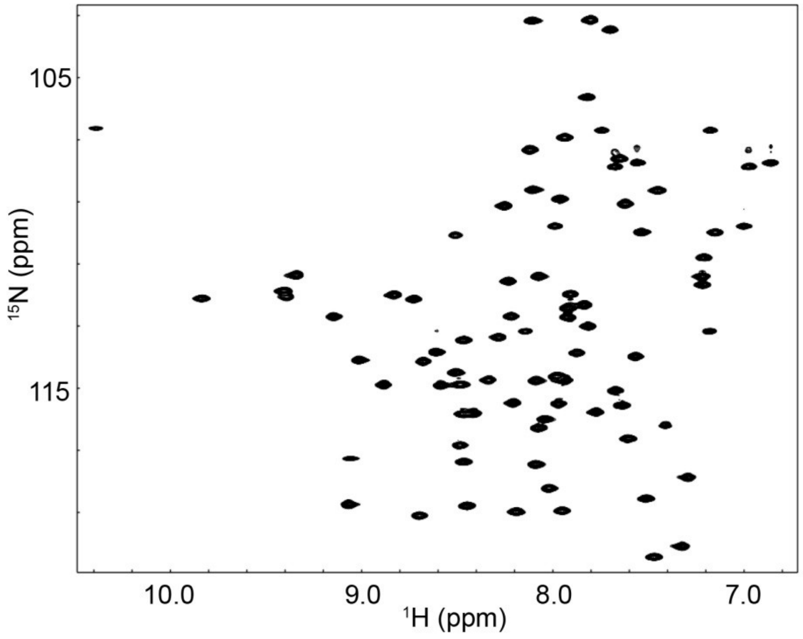

Holo-PaACP
(wild type)

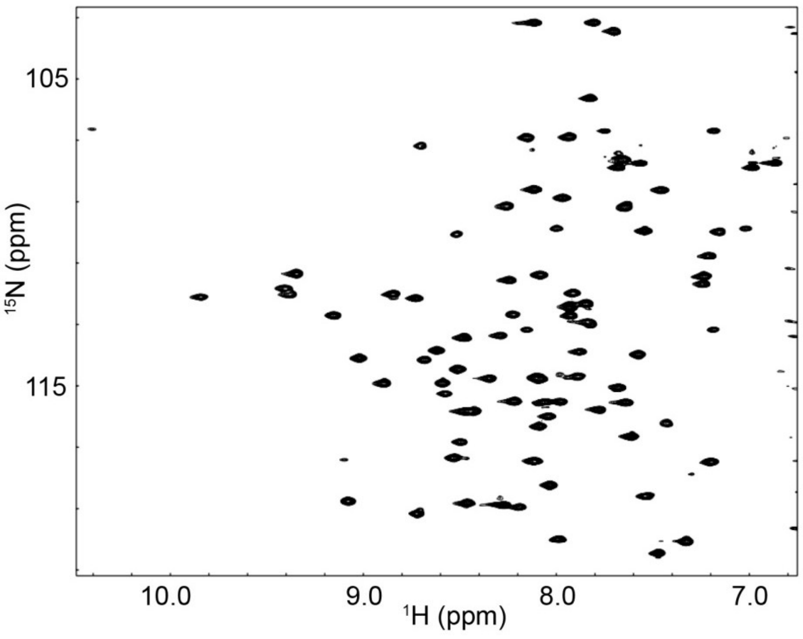

Figure S2. ^{1}H,^{15}N HSQC NMR spectra of purified apo- and holo-PaACP showing that both proteins are over 90% pure. A few minor peaks in the spectrum of holo-PaACP reflect the presence of <10% of apo-PaACP. Such minor peaks are not seen in the HSQC spectrum of apo-PaACP.

A. Apo-FnACP S52A_ Lys-rich F5W Magainin 2

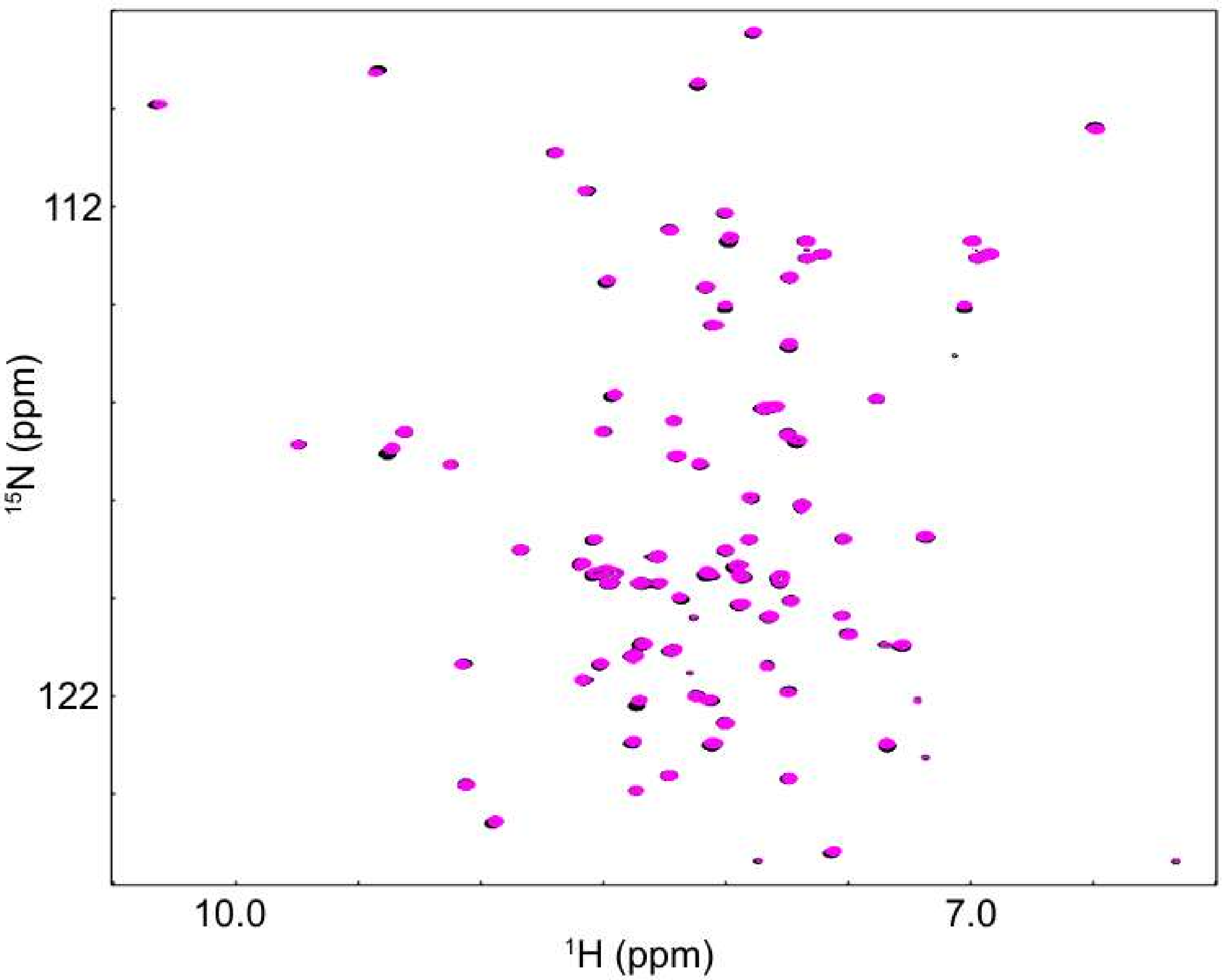

B. Apo PaACP S37A_Lys-rich F5W Magainin 2

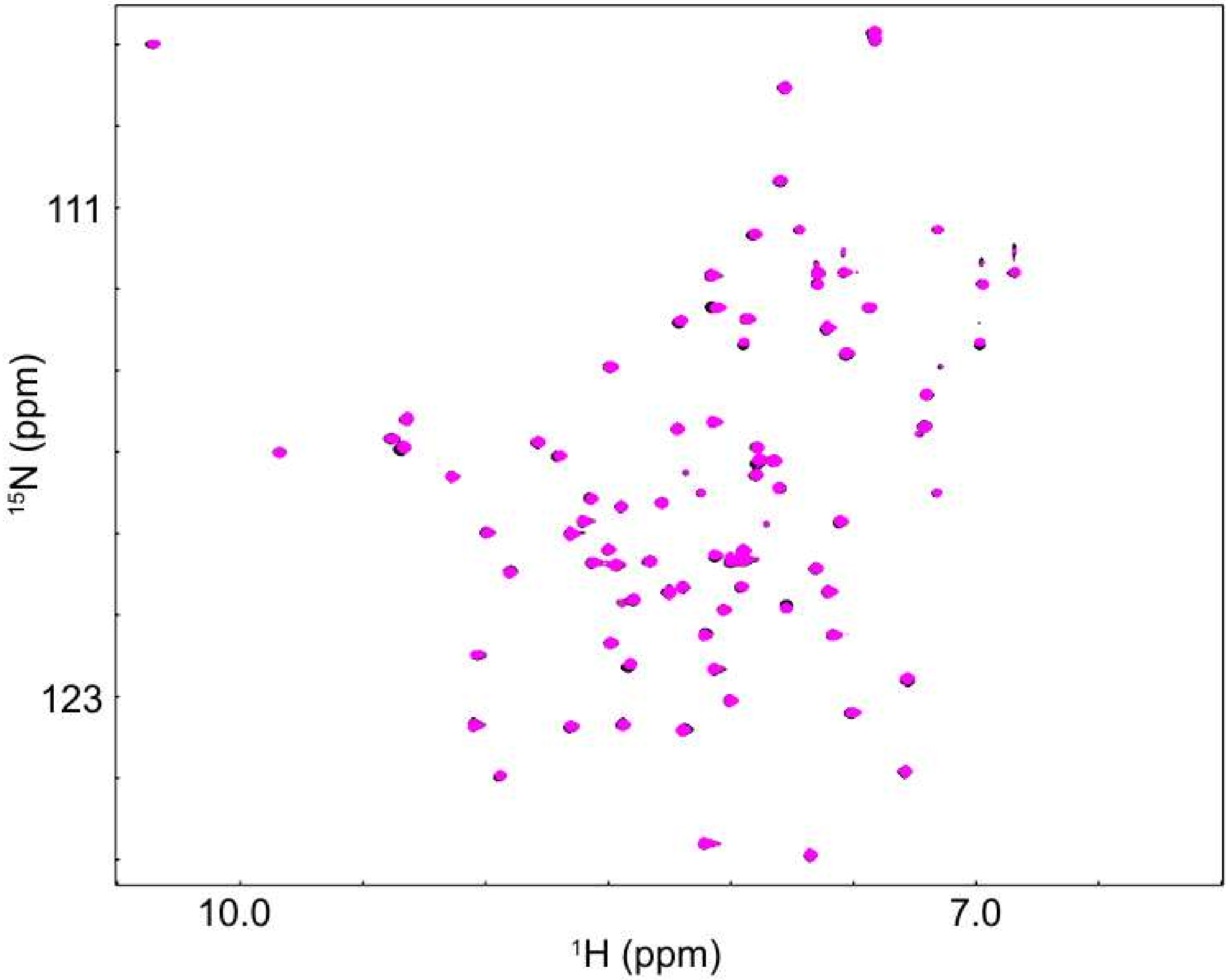

Figure S3. NMR titrations recorded for (A) apo-FnACP S52A and (B) apo-PaACP S37A in the presence of Lys-rich F5W magainin 2. The spectrum of ACP alone is shown in black, and the titration between ACP with Lys-rich F5W magainin 2 at 1:1 ratio spectrum is shown in magenta. In all cases the peaks fully overlap, suggesting that this AMP does not bind to the two proteins.

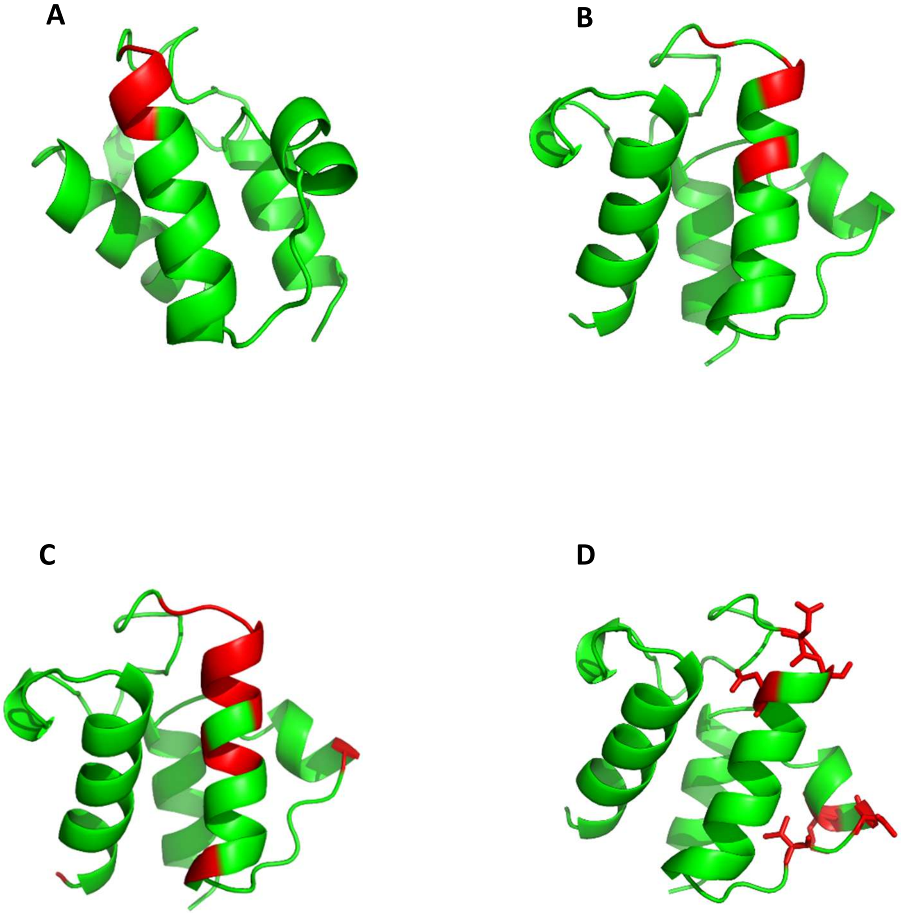

Figure S4. Residues (red) of ACPs that are involved in the interaction with different AMPs are mapped on the model structure of apo-ACP from *P. aeruginosa* FAS system. The SWISS-MODEL homology program was used to build the model using the solution structure of *V. harveyi* (2L0Q) as a template. A. LL-37, B. tritrpticin, C. indolicidin and D. IDR-1018. Please note, the proteins are not depicted in the same position but helix II is most clearly shown in all cases. The DSL site is located near the top of helix II.

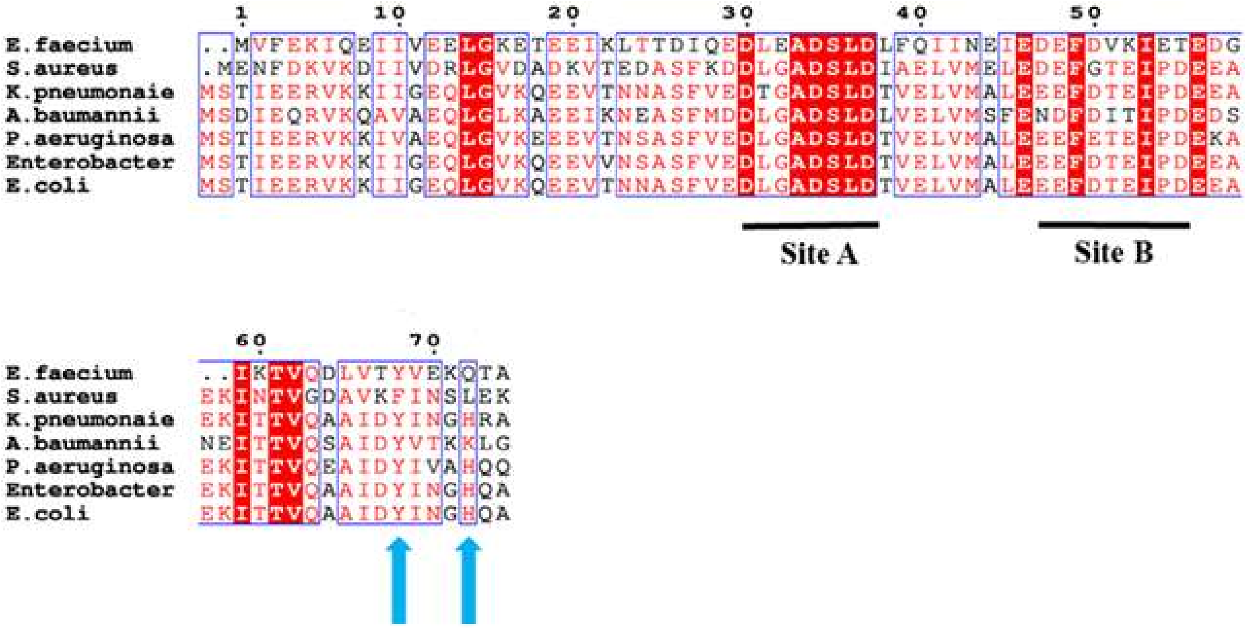

Figure S5. Amino acid sequence alignment of the FAS-ACP proteins from the ESKAPE(E) organisms, that are responsible for most hospital-acquired multidrug-resistant bacterial infections. The location of the two divalent metal ion binding sites are indicated. Site A and site B refer to the two binding sites for divalent metal ions. The position of the conserved Tyr (Y) and His (H) that are involved in the stabilizing cation- π interaction are indicated by the blue arrows.

Table S1. The antimicrobial peptides used in this study along with their properties are listed. The relative hydrophobicity of each AMP was experimentally determined by using a standard HPLC protocol (see Ishida et al (2016) J. Am. Chem. Soc.138,11318-11326). For a review on structural properties, see Nguyen et al (2011), Trends Biotechnol. 29, 464-472.

AMPs	Sequence	Structure	Length	Net Charge	R	K	W	Relative Hydrophobicity (% acetonitrile)
LL-37 (pI 10.61)	LLGDFFRKSKEK IGKEFKRIVQRI KDFLRNLVPRT ES	α-helix	37	+6	5	6	0	ND
Melittin (pI 12.02)	GIGAVLKVLTT GLPALISWIKRK RQQ	α-helix	26	+5	2	3	1	39.50
F5W-magainin 2 (pI 10.00)	GIGKWLHSAKK FGKAFVGEIMN S-NH_2	α-helix	23	+4	0	4	1	32.10
Tritrpticin (pI 12.48)	VRRFPWWWPF LRR-NH_2	Turn	13	+5	4	0	3	34.45
Indolicidin (pI 12.01)	ILPWKWPWWP WRR-NH_2	Turn	13	+4	2	1	5	34.40
Puroindoline A (pI 11.17)	FPVTWRWWKW WKG-NH_2	Turn	13	+4	1	2	5	32.40
Lactoferricin B (pI 11.84)	FKCRRWQWRM KKLGAPSITCVR RAF	β-hairpin	25	+8	5	3	2	33.50
IDR-1018 (pI 12.48)	VRLIVAVRIWRR -NH_2	α-helix	12	+5	4	0	1	31.20

Table S2. List of enzymes involved in interactions with different forms of ACPs during fatty acid biosynthesis. The abbreviated names of enzymes are within the parenthesis, such as malonyl coenzyme A: acyl carrier transacylase (MCAT), ketoacyl synthase (KS), ketoacyl reductase (KR), dehydratase (DH), enoyl reductase (ER), thioesterase (TE), and acyl carrier protein synthase (ACPS). This table also highlights the methods, which are used to probe the interaction and residues of ACPs participated in the interaction. The terminology crypto-ACP indicates the ACP with modified pantetheine analog. (Most of this information can be found in the RCSB PDB and associated references. Further details can be obtained from the authors upon request)

Enzyme	Organism	ACP	Methods	Validation	Residues of ACP
MCAT	*H. pylori*	Apo-	Modeling	SPR & GST-pull-down	E13, N16, D35, L37, V39, V40, E41, I43, M44, E47, D56, E57, A59, E60
	S. coelicolor	Holo-	Modeling	Mutation, Enzyme Kinetics & NMR	D40, E46, E52, E53
KS	*E. coli*	Apo-	Modeling	Mutation & Enzyme kinetics	R6, E13, S36, E41, M44, A45, E47, E48, E49
KR	*E. coli*	Holo-	NMR	-	L15, D35, S36, D38, T39, L42, V43, A45, E48, T52, E53, I54, Q66
DH	*E. coli*	Crypto-<br>Holo-<br>Acyl-	X-ray<br>NMR	-	D35, D38, V40, E41, E47, T52, A59, E60, T63, A68
	H. pylori	Holo	X-ray	-	D35, D38, E41, M44, E47
ER	*E. coli*	Acyl-	X-ray<br>MD simulation	Mutation & Enzyme kinetics	Q14, D35, D38, E41, E48
TE	*C. reinhardtii*	Crypto-	Modeling	Activity based crosslinking	Helix II (Specific residues not mentioned)
ACPS	*B. subtilis*	Apo	X-ray	-	D35, D38, E41, D48, I54, D56, E60
